# Colonization Patterns, Phenology and Seasonal Abundance of the Nearctic Leafhopper *Erasmoneura vulnerata* (Fitch), a New Pest in European Vineyards

**DOI:** 10.3390/insects11110731

**Published:** 2020-10-26

**Authors:** Carlo Duso, Giulia Zanettin, Pamela Gherardo, Giulia Pasqualotto, Damiano Raniero, Filippo Rossetto, Paola Tirello, Alberto Pozzebon

**Affiliations:** Department of Agronomy, Food, Natural Resources, Animals and Environment, University of Padova, Viale dell’Università 16, Agripolis, Legnaro, 35020 Padova, Italy; giuliazanettin22@gmail.com (G.Z.); pamela.gherardo@studenti.unipd.it (P.G.); giulia.pasqualotto.2@studenti.unipd.it (G.P.); damiano.raniero@studenti.unipd.it (D.R.); filippo.rossetto.1@studenti.unipd.it (F.R.); paola.tirello@unipd.it (P.T.); alberto.pozzebon@unipd.it (A.P.)

**Keywords:** *Erasmoneura vulnerata*, *Vitis vinifera*, edge effect, seasonal abundance, phenology, vineyard management

## Abstract

**Simple Summary:**

The leafhopper *Erasmoneura vulnerata* (Fitch) (Hemiptera: Cicadellidae) is native to North America, and was found in Europe for the first time (north-eastern Italy) in 2004 where it remained a minor pest of grapevine for more than ten years. Recently, its importance increased in commercial vineyards located in north-eastern Italy, where outbreaks of *E. vulnerata* populations with severe leaf symptoms were observed despite insecticide applications. Information on its biology and ecology is needed for the development of management strategies. Here, we investigated the phenology and seasonal abundance of *E. vulnerata* in commercial vineyards. We found that *E. vulnerata* can complete three generations per growing season. Vineyard colonization by overwintered adults showed a clear edge effect, suggesting an effect of overwintering sites (e.g., rural buildings and hedgerows) adjacent to vineyards. The impact of natural enemies on pest populations appeared to be limited and mostly related to egg parasitoids. Organic vineyards were more heavily infested by *E. vulnerata* compared to conventional vineyards, likely due to the low effectiveness of natural insecticides typically used in the former farms. The results generated by this study provide implications for the management of this pest in European vineyards.

**Abstract:**

The Nearctic leafhopper *Erasmoneura vulnerata* (Fitch), detected in Europe for the first time (north-eastern Italy) in 2004, has remained a minor pest of grapevine for more than 10 years. The first outbreaks of *E. vulnerata* were reported in 2016 in commercial vineyards located in north-eastern Italy. High population densities and severe leaf symptoms (i.e., leaf discoloration and fall) were observed in late summer despite the application of insecticides. Investigations were carried out from 2017 to 2019 in 10 vineyards located in Veneto region (Vicenza and Verona provinces) to shed light on the seasonal abundance of *E. vulnerata* on different *Vitis vinifera* cultivars. Pest phenology was studied in six vineyards where the impact of insecticides was minimal. *Erasmoneura vulnerata* completed three generations in each of the growing seasons. Vineyard colonization by overwintered adults showed a clear edge effect, suggesting the influence of overwintering sites (e.g., rural buildings and hedgerows) in vineyard margins. The impact of natural enemies on pest populations appeared to be limited and mostly related to egg parasitoids. Organic vineyards were more heavily infested by *E. vulnerata* compared to conventional vineyards, likely due to the minimal efficacy of natural insecticides typically used in the former farms.

## 1. Introduction

The leafhopper *Erasmoneura vulnerata* (Fitch) (Hemiptera: Cicadellidae) is native to Northern America, and has belonged to the genus *Erythroneura* for an extended period of time [1,2,3]. In 2006, Dietrich and Dmitriev [4] placed this species in the genus *Erasmoneura*, that comprises 11 taxa including *Erasmoneura variabilis* (Beamer), a key pest in California vineyards [4,5,6,7,8]. In its native range, *E. vulnerata* was collected on American and European grapevine species, as well as on alternative hosts, primarily *Parthenoccissus quinquefolia* (L.) Planch, *Ilex decidua* Walter and *Cercis canadensis* L. [8]. In some publications, *E. vulnerata* has been reported as being a severe pest of grapevine in the USA [9,10], but more recent investigations have shown that this species was rarely dominant in leafhopper communities occurring in American vineyards (e.g., [11,12,13]).

*Erasmoneura vulnerata* was recorded in Europe for the first time in 2004 (Veneto region, north-eastern Italy) [14]. Some aspects of *E. vulnerata* behavior in Italy were described by Girolami et al. [15]. Nymphs and adults feed on leaf mesophyll, and feeding sites appear as pale speckled areas. When population densities increase, feeding areas overlap and involve the entire leaf. An additional symptom is represented by the presence of black excrements on the foliage. Heavily infested leaves may dislodge prematurely [12,14]. Information regarding damage assessment and economic thresholds is lacking. Field and semi-field studies carried out on pesticide-free vines suggested that *E. vulnerata* could develop two or three generations per year [16,17]. Pesticide use was likely the most important factor influencing the spread of *E. vulnerata* into newly invaded areas and its abundance in vineyards. On untreated vines, *E. vulnerata* dominated over the native leafhoppers *Empoasca vitis* (Göethe) and *Zygina rhamni* Ferrari. In contrast, the opposite situation was reported in commercial vineyards where insecticides were applied to control *Scaphoideus titanus* Ball and other pests [15].

Since 2005, *E. vulnerata* has spread to new areas both in Italy and Slovenia [16,18], but populations remained at low-density levels in vineyards. This situation changed in 2016, when outbreaks were detected in commercial (*Vitis vinifera*) vineyards located in the Veneto region (Treviso and Vicenza provinces). In late summer, symptoms caused by this leafhopper were spread on more than 90% of the canopy, and population densities exceeded 10 nymphs per leaf in some vineyards [19]. Surprisingly, infestations were detected both in conventional and organic vineyards despite the application of insecticides. Recently, *E. vulnerata* has been recorded in southern Switzerland [20] and Serbia [21].

This new scenario prompted the need for in-depth investigations on colonization patterns, phenology, and abundance in areas where the insect has become a significant pest in vineyards. Previous observations of *E. vulnerata* phenology were carried out on untreated *Vitis labrusca* vineyards, and *V. vinifera* cultivars were used for semi-field experiments [17]. These observations also suggest that there may be an effect of overwintering sites at the vineyard borders on the colonization patterns of *E. vulnerata* like for other leafhoppers [22] Therefore, the phenology of *E. vulnerata* was studied in some *V. vinifera* vineyards, and the effect of field margins on adult *E. vulnerata* colonization patterns in vineyards was also investigated. Vineyard management (organic vs. conventional) was also considered as a factor influencing the seasonal abundance of *E. vulnerata*.

## 2. Materials and Methods

### 2.1. Study Sites

Investigations of adult *E. vulnerata* colonization patterns (i.e., effects of vineyard margins) were carried out in four vineyards per year, from 2017 to 2019 (Table 1). In these vineyards, located in the Vicenza and Verona provinces, the occurrence of *E. vulnerata* had been reported in the season preceding the study. Vineyards selected for the study on colonization patterns were not treated with insecticides from bud break to blossom when observations were completed. Some of these vineyards were not considered during investigation of *E. vulnerata* phenology because of insecticide applications during the summer; therefore, leafhopper phenology was investigated in two vineyards per year that received limited or no insecticide applications from 2017 to 2019 (Table 1).

### 2.2. Effects of Distance from Vineyard Margins on Erasmoneura vulnerata Colonization Patterns

**Figure 1:**
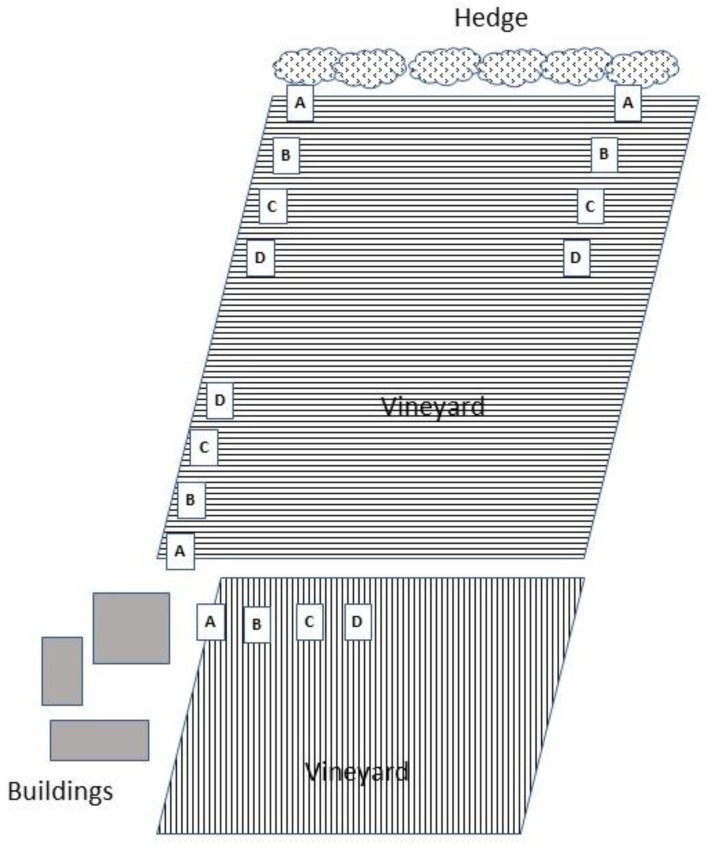
Example of trap position in the study on the effects of distance from vineyard margins on *E. vulnerata* colonization patterns (AC vineyard in 2017).

Observations carried out before bud break revealed the occurrence of many *E. vulnerata* adults on hedges and shrubs (e.g., associated with rural buildings, suggesting that leafhoppers overwintered at these sites). In particular, we observed *E. vulnerata* adults on *Parthenocyssus tricuspidata, Rosa* spp. and *Prunus* spp. The presence of hedgerows contiguous to vineyards was also considered to be a factor potentially affecting *E. vulnerata* overwintering [13], and thus the role of vineyard margins in the leafhopper’s colonization was investigated. In 2017, four transects, each comprising four yellow sticky traps, were arranged at increasing distances from rural buildings and hedgerows contiguous to AC vineyard. Four positions were identified for trap placement: A (first vineyard row close to rural buildings or hedgerows), B, C, and D (20, 40, and 60 m from position A, respectively) (Figure 1). An additional five transects were arranged in AO and LO1 farms for a total of nine transects in the 2017 growing season. Observations were carried out from April to June, focusing on the most common leafhopper species, i.e., *E. vulnerata*, *E. vitis* and *Z. rhamni*; they were identified using keys published by Dmitriev [8] and Vidano [23]. Traps were analyzed in the laboratory using a stereomicroscope and were replaced every 7–10 days. In 2018 and 2019, a total of four transects (one per vineyard) were planned. Sampling was carried out from April to June, adopting the experimental approach from 2017.

### 2.3. Phenology of Erasmoneura vulnerata

In 2017, leaf sampling was regularly carried out on insecticide-free plots of AC vineyard (cv. Cabernet Sauvignon), while in AO vineyard (cvs. Merlot, Glera, Pinot gris, Incrocio Manzoni 6013) pyrethrins were only applied in late July to control *S. titanus*. In AC vineyard, a total of 30 leaves were inspected every 7–10 days in the laboratory using a dissecting microscope. Individual leafhoppers were counted considering their identity and age, and age was subdivided into three categories (I–II instar nymphs, III–V instar nymphs, adults). The occurrence of potential predators or parasites of leafhoppers on leaf samples was also evaluated, as well as egg parasitoid emergence holes. Data from leaf samplings were coupled with data from adults captured on four yellow sticky traps placed within the grapevine canopy. Traps were analyzed in the laboratory under a dissecting microscope and were renewed every sampling date. The same procedure was applied in AO vineyard, where a total of 60 leaves (15 leaves per cultivar) were analyzed. In 2018, 30 leaves were collected in LO2 and MO vineyards approximately every 10–15 days and were then analyzed in the laboratory following the previously described procedures. Four yellow sticky traps were also placed in each vineyard. Mineral oils were applied in MO vineyard. The same approach was applied in 2019 in LO1 and MO vineyards. Pyrethrins were applied in LO1 vineyard, and kaolin in MO vineyard.

### 2.4. Effects of Vineyard Management on Erasmoneura vulnerata Seasonal Abundance

In 2018, the effect of vineyard management (organic vs. conventional) on *E. vulnerata* abundance was tested comparing four organic and four conventional vineyards located in the Vicenza and Verona provinces (at Alonte, Lonigo, Monteforte d’Alpone, Roncà). We selected AO, LO2, MO, and MO2 among organic vineyards (MO2 vineyard was located at Monteforte D’Alpone and comprised Garganega cultivar), while AC, LC, MC, and RC were selected among conventional vineyards (RC vineyard was located at Roncà and comprised Garganega cultivar). In each vineyard, 30 leaves and four yellow sticky traps were analyzed in the laboratory approximately every two weeks, from May to September. Leafhoppers were identified at species level focusing on *E. vulnerata*, *E. vitis,* and *Z. rhamni*. The number of egg parasitoid (Hymenoptera: Mymaridae) emergence holes was also taken into account to calculate parasitism rates. These values were calculated by dividing the number of parasitoid emergence holes by the sum of the nymph hatching holes and the parasitoid emergence holes, expressed as a percentage. A number of emerging cages [20] were used to isolate adult parasitoids and identify them to the species level using molecular markers. Using the salting-out protocol [24], mitochondrial DNA was extracted from 15 samples of the parasitoid *Anagrus* spp., which were reared in the laboratory from grapevine leaves infested by *E. vulnerata.* A fragment of the cytochrome c oxidase subunit 1 (COI) was amplified and sequenced using the procedure described in Martinez-Sañudo et al. [25].

### 2.5. Statistical Analyses

Data from these experiments aimed at investigating the effects of distance from vineyard margins on leafhopper colonization and were analyzed using repeated measures linear mixed model with the MIXED procedure of SAS^®^ (ver. 9.3; SAS Institute Inc., Cary, NC, USA). Distance from the margin, time of sampling, and their interaction were considered as sources of variation in the model and were tested using an F test (α = 0.05). The transect was considered as a random effect in the model. Pairwise comparisons of catches placed at different distances were performed using Tukey’s test (α = 0.05) on the least-square means. The degrees of freedom were estimated with Kenward–Roger method. Prior to the analysis, data were checked for model assumptions. The model was run on data transformed to log (*n* + 1), while untransformed data are shown in figures.

Effects of vineyard management on the abundance of *E. vulnerata*, *E. vitis* and *Z. rhamni* adults on traps, nymphs on leaves, as well as on the parasitism rate by the Mymaridae were analyzed separately with a repeated measures linear mixed model with the MIXED procedure of SAS^®^ (ver. 9.3; SAS Institute Inc., Cary, NC, USA). In this analysis, vineyard management (organic vs. conventional), time of sampling, and their interaction were considered as sources of variation and were tested with an F test (α = 0.05). Comparisons between vineyard management on each date were performed using Tukey’s test (α = 0.05) on the least-square means. The degrees of freedom were estimated using the Kenward–Roger method. Prior to the analysis, data were checked for model assumptions, and leafhoppers captures were transformed to log (*n* + 1), while arcsin of the square root was applied to the parasitism rate data. Untransformed data are shown in the figures.

## 3. Results

### 3.1. Effects of Distance from Vineyard Margins on Erasmoneura vulnerata Colonization Patterns

#### 3.1.1. 2017

**Figure 2:**
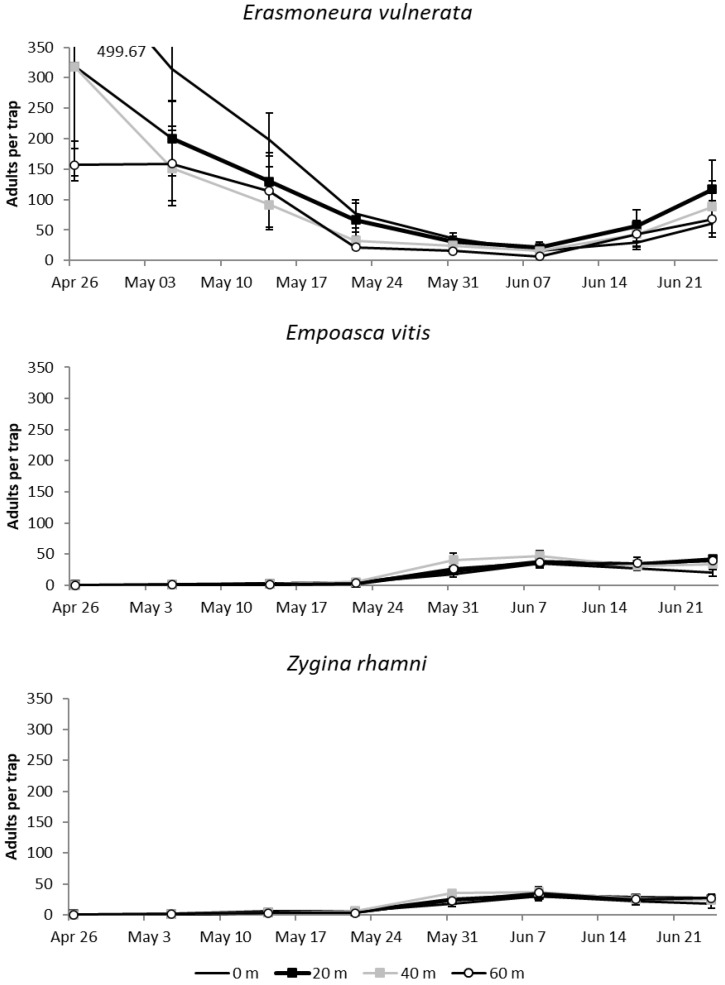
Mean (± std. err.) number of adult *E. vulnerata*, *E. vitis* and *Z. rhamni* catches on traps placed at increasing distances from the vineyard margins in the spring of 2017.

Adult *E. vulnerata* catches were abundant in late April when adults moved inside the vineyards, while catch numbers declined in May and slightly increased in June (F = 24.15; d.f. = 7, 179; *p* < 0.0001; Figure 2). Differences among treatments (increasing distances from the margin) were significant (F = 4.11; d.f. = 3, 30.6; *p* = 0.014) and the most abundant catch numbers were detected at the vineyard margin (0 m vs. 40 m: t = 3.01; d.f. = 30.6; *p* = 0.025; 0 m vs. 60 m: t = 2.94; d.f. = 30.7; *p* = 0.029). There were no differences between catches on traps located at 0 m and 20 m (t = 1.41; d.f. = 30.6; *p* = 0.506). The remaining comparisons were not significant (20 m vs. 40 m: t = 1.6; d.f. = 30.5; *p* = 0.392; 20 m vs. 60 m: t = 1.54; d.f. = 30.6; *p* = 0.426; 40 m vs. 60 m: t = −0.06; d.f. = 30.6; *p* = 0.999). Empoasca vitis and Zygina rhamni catch numbers were much lower, and reached the highest levels in June (Figure 2). The effect of margins on number of adult catches was not significant (F = 1.56; d.f. = 3, 60; *p* = 0.208; F = 0.55; d.f. = 3, 53.2; *p* = 0.653 for *E. vitis* and *Z. rhamni*, respectively).

#### 3.1.2. 2018

**Figure 3:**
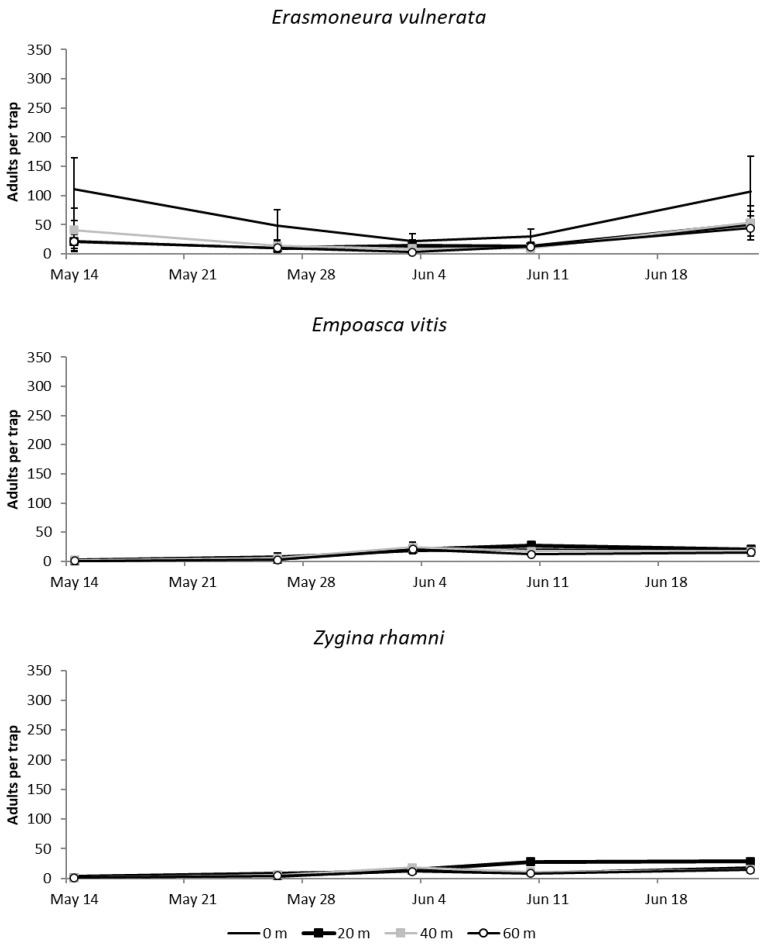
Mean (± std. err.) number of adult *E. vulnerata*, *E. vitis* and *Z. rhamni* catches on traps placed at increasing distances from the vineyard margins in the spring of 2018.

Numbers of adult *E. vulnerata* catches were higher than those of *E. vitis* and *Z. rhamni* in early spring of 2018 (Figure 3). Adult *E. vulnerata* numbers fluctuated over the sampling dates leading to a significant effect of time (F = 9.01; d.f. = 4, 40.4; *p* < 0.0001). Differences among treatments (increasing distances from the margin) were also significant (F = 7.45; d.f. = 3, 9.88; *p* = 0.0068) and the number of catches was higher at the vineyard margin (0 m vs. 20 m: t = 3.56; d.f. = 9.88; *p* = 0.023; 0 m vs. 40 m: t = 3.82; d.f. = 9.88; *p* = 0.015; 0 m vs. 60 m: t = 4.12; d.f. = 9.88; *p* = 0.009). The remaining comparisons were not significant (20 m vs. 40 m: t = 0.26; d.f. = 9.88; *p* = 0.994; 20 m vs. 60 m: t = 0.56; d.f. = 9.88; *p* = 0.994; 40 m vs. 60 m: t = 0.31; d.f. = 9.88; *p* = 0.989). The number of *Z. rhamni* and *E. vitis* catches reached the highest levels in June (Figure 2), and the effect of margins on their adults was not significant (F = 0.87; d.f. = 3, 15.8; *p* = 0.477; F = 1.56; d.f. = 3, 16.2; *p* = 0.236).

#### 3.1.3. 2019

**Figure 4:**
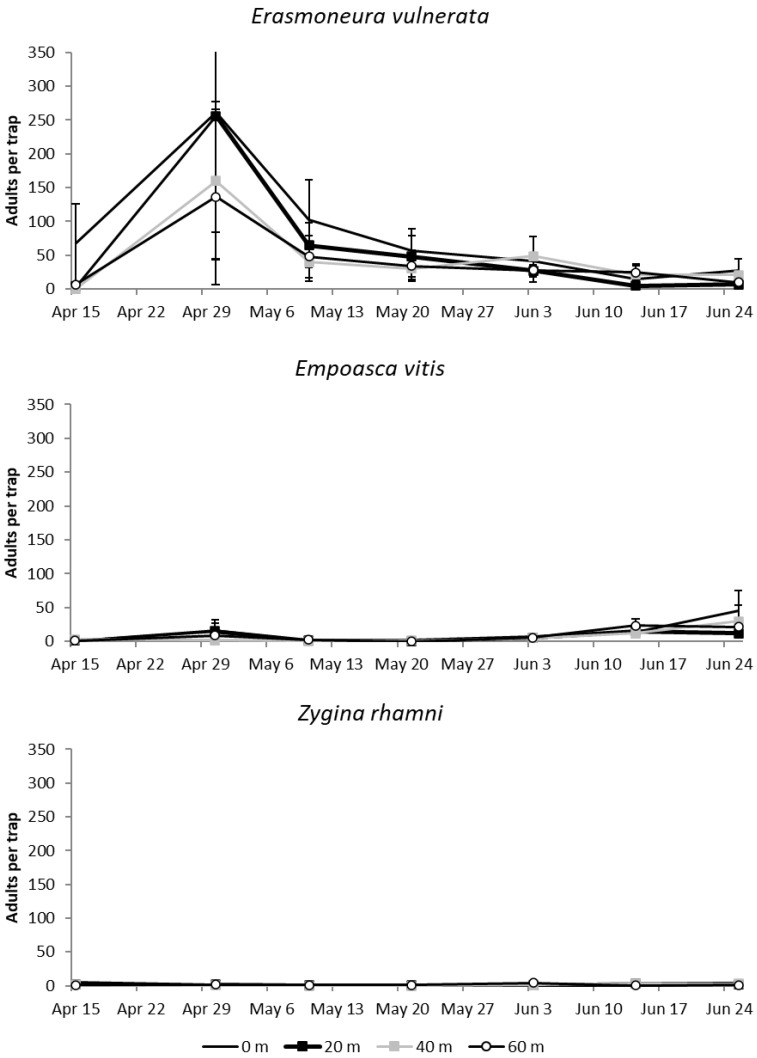
Mean (± std. err.) number of adult *E. vulnerata*, *E. vitis* and *Z. rhamni* catches on traps placed at increasing distances from the vineyard margins in the spring of 2019.

The number of *E. vulnerata* catches increased in April when adults moved inside the vineyards, while later catches declined in number (F = 11.99; d.f. = 6, 78.3; *p* < 0.0001; Figure 4). Differences among treatments were significant (F = 5.19; d.f. = 3, 21.6; *p* = 0.044), and the highest number of catches were detected at the vineyard margin (0 m vs. 20 m: t = 2.12; d.f. = 21.6; *p* = 0.045; 0 m vs. 40 m: t = 2.71; d.f. = 21.6; *p* = 0.013; 0 m vs. 60 m: t = 2.59; d.f. = 21.6; *p* = 0.017). The remaining comparisons were not significant (20 m vs. 40 m: t = 0.59; d.f. = 21.6; *p* = 0.564; 20 m vs. 60 m: t = 0.46; d.f. = 21.6; *p* = 0.648; 40 m vs. 60 m: t = −0.12; d.f. = 21.6; *p* = 0.903). The number of *E. vitis* and *Z. rhamni* catches were much lower (Figure 3), and the effect of margins on them was not significant (F = 0.19; d.f. = 3, 23.2; *p* = 0.9; F = 0.1; d.f. = 3, 99.7; *p* = 0.961 for *E. vitis* and *Z. rhamni*, respectively).

### 3.2. Phenology of Erasmoneura vulnerata

#### 3.2.1. 2017

**Figure 5:**
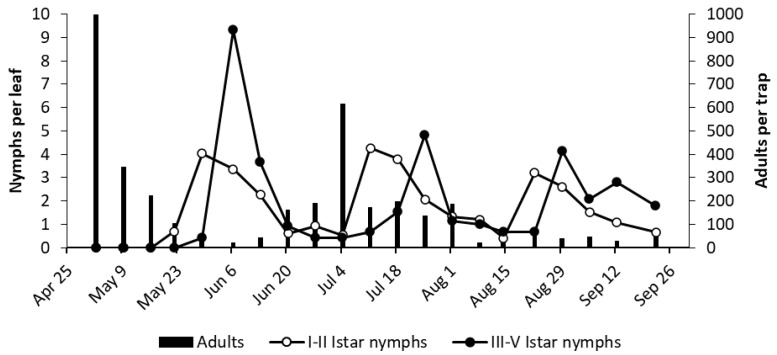
The phenology of *E. vulnerata* in AC vineyard in the growing season of 2017.

At bud break in 2017 (April 10th), high numbers of *E. vulnerata* adults were caught on yellow sticky traps of the selected vineyards (Figure 5). The number of *E. vitis* and *Z. rhamni* catches was much lower (data not shown). In subsequent weeks, the number of *E. vulnerata* catches declined. In AC vineyard, early instar nymphs of *E. vulnerata* were first detected in the second half of May, and their densities peaked in late May (Figure 5). Older nymph numbers peaked in early June. The abundance of early instar nymphs showed two additional peaks in early July and late August, followed by those of older nymphs. The number of adult catches showed two clear peaks following those of older nymphs in early July and early August. Their numbers declined in late summer. The data strongly suggest the development of three generations. Densities of other leafhopper taxa in leaf samples were negligible (data not shown).

**Figure 6:**
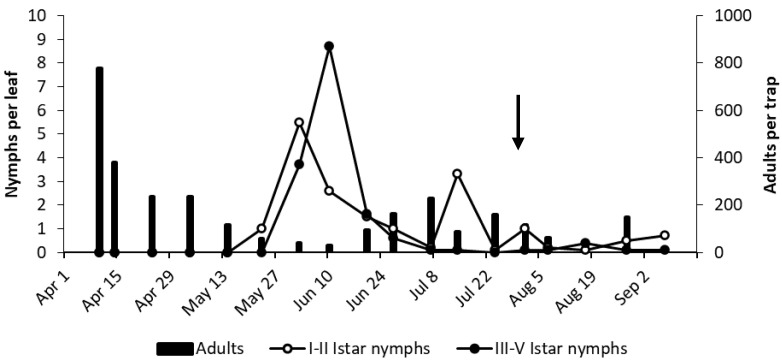
The phenology of *E. vulnerata* in AO vineyard in the growing season of 2017. Arrow indicates pyrethrins treatment.

In AO vineyard, observations were carried out on four different varieties, and the phenology of *E. vulnerata* reported in Figure 6 was obtained by combining these data. Early instar nymphs were detected from late May, and their densities peaked in early June, followed by an increase in older nymphs and adults; therefore, the first generation developed in a similar way to that of the AC vineyard. A new increase in early instar nymphs in the first half of July was compatible with the start of the second generation; however, in contrast with the situation reported in AC vineyard, older nymphs reached negligible densities in July. This was likely due to the application of pyrethrins to control *S. titanus*. A slight increase in early instar nymphs detected in late August and early September could indicate the onset of the third generation. The occurrence of other leafhoppers in this vineyard was negligible (data not shown).

#### 3.2.2. 2018

**Figure 7:**
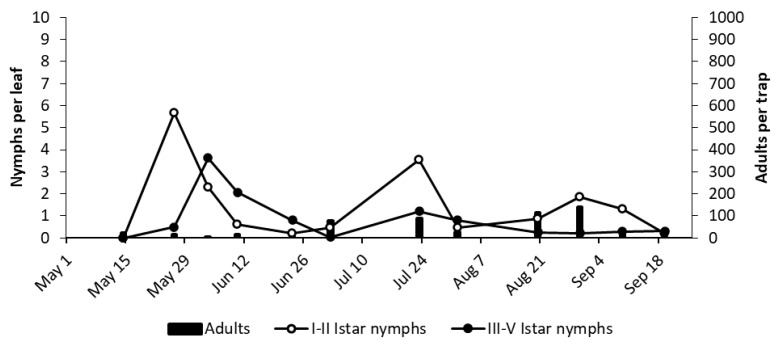
The phenology of *.E. vulnerata* in MO vineyard in the growing season of 2018.

**Figure 8:**
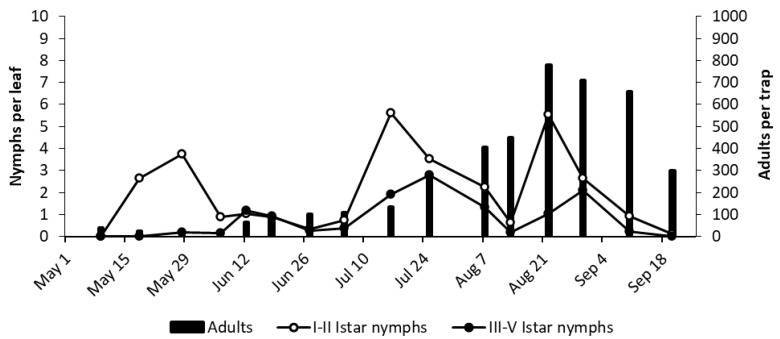
The phenology of *E. vulnerata* in LO2 vineyard in the growing season of 2018.

At bud break in 2018, *E. vulnerata* adults were abundant; however, when regular sampling started in early May, the number of adult catches in the selected vineyards (MO and LO2) were relatively low (Figure 7 and Figure 8). Early instar nymphs were first detected in mid-May, and their numbers peaked in late May, followed by peaks of older nymphs (Figure 7 and Figure 8). The dynamics of early instar nymphs showed additional peaks in the second half of July and in late August to early September, whereas older nymphs peaked in the same or in subsequent sampling dates. Adult numbers (caught on traps) did not follow homogenous patterns, but, in most cases, reached higher numbers in August (Figure 7 and Figure 8). The occurrence of other leafhoppers on traps, as well on leaf samples, was much lower (data not shown).

#### 3.2.3. 2019

**Figure 9:**
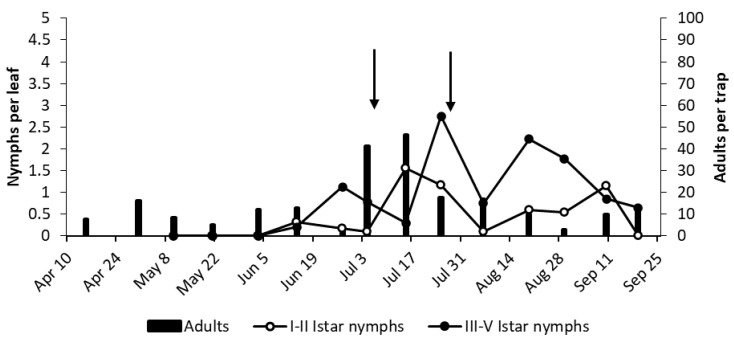
The phenology of *E. vulnerata* in MO vineyard in the growing season of 2019. Arrows indicate pyrethrins treatments.

**Figure 10:**
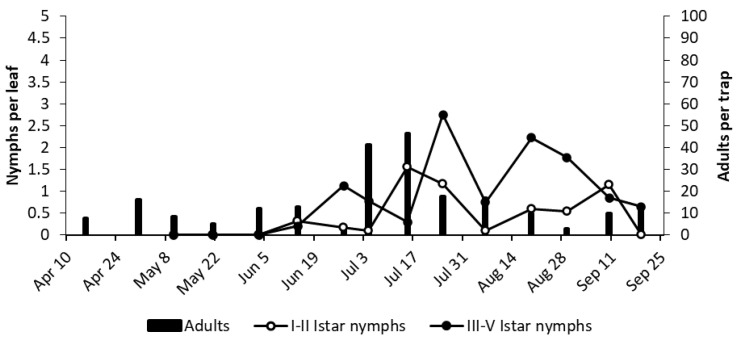
The phenology of *E. vulnerata* in LO1 vineyard in the growing season of 2019.

The number of adult *E. vulnerata* catches was low in the spring of 2019, likely due to the occurrence of frequent rainfall (Figure 8 and Figure 9). Early instar nymphs peaked in mid-June, late July, and the first half of September in both vineyards (Figure 9 and Figure 10). Excluding late summer, the dynamics of older nymphs and adults were consistent with that of early nymphs (Figure 8 and Figure 9). Low densities of *E. vitis* and *Z. rhamni* were recorded in both vineyards.

### 3.3. Natural Control

A number of Heteroptera (*Malacocoris chlorizans* Panzer and *Orius* spp. in particular), as well as Neuroptera (e.g., *Chrysoperla carnea* Stephens), were seldom observed preying on leafhopper nymphs in leaf samples. Nymphs of *Allothrombium* sp. (Acari Prostigmata) were also detected feeding on leafhopper nymphs. Parasitism by Hymenoptera Mymaridae was ascertained in all vineyards through the observation of emergence holes left by parasitoid adults on leaf veins. Among the Mymaridae that emerged from grapevine leaves in the laboratory, 14 individuals belonged to *Anagrus parvus* Soyka sensu Viggiani [26] and only one belonged to *Anagrus atomus* (Linnaeus) [27].

### 3.4. Effect of Vineyard Management

**Figure 11:**
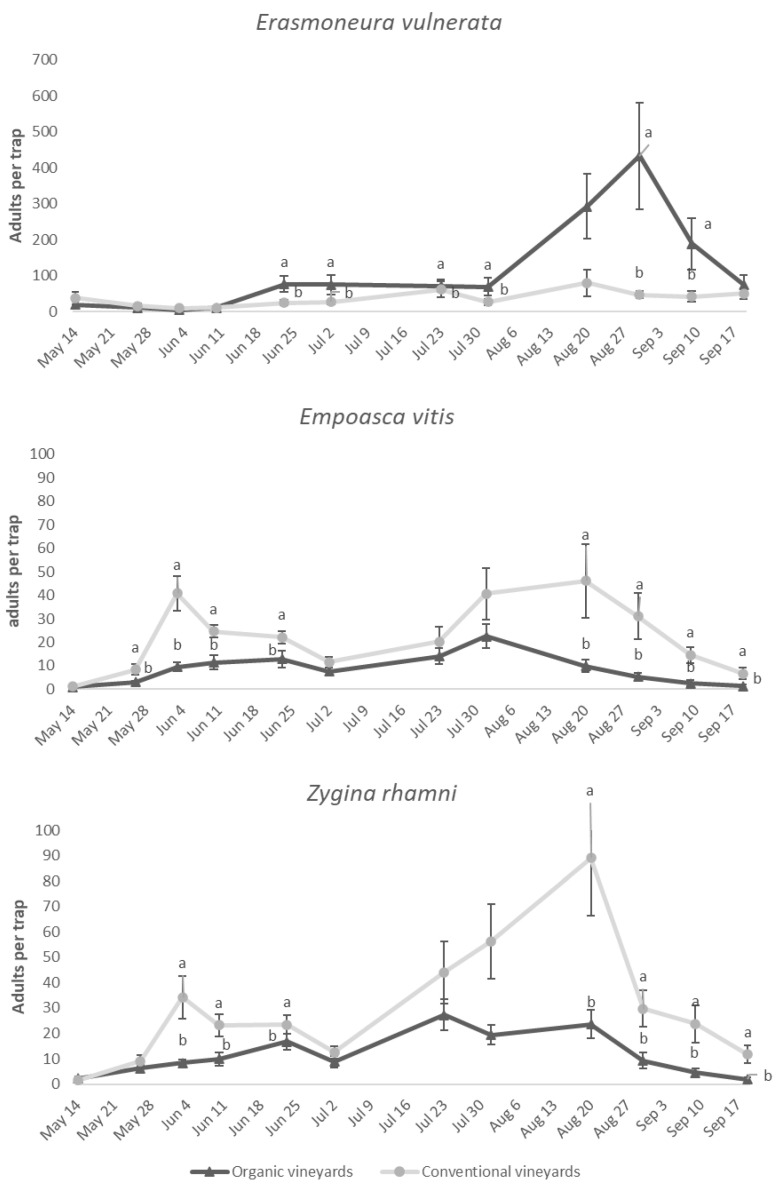
Mean (± std. err.) number of adult *E. vulnerata*, *Z. rhamni* and *E. vitis* catches on traps placed in organic and conventional vineyards. Different letters indicate significant differences based on Tukey’s test on least square means (α = 0.05) of the number of adults caught on each date.

The number of adult *E. vulnerata* catches was higher in organic vineyards (F = 6.05; d.f. = 1, 37.5; *p* = 0.018) and differed by the time of sampling and by the interaction vineyard management * time (F = 17.00; d.f. = 11, 308; *p* < 0.0001; F = 2.56; d.f. = 11, 318; *p* = 0.004, respectively). The number of adult *E. vulnerata* catches was higher in organic vineyards on multiple sampling dates (Figure 10). Vineyard management affected the number of adult *E. vitis* catches (F = 27.11; d.f. = 1, 61.08; *p* < 0.0001) as well as the effect of time and the interaction vineyard management * time (F = 19.33; d.f. = 11, 313; *p* < 0.0001; F = 3.47; d.f. = 11, 313; *p* = 0.001). In particular, the number of *E. vitis* catches was higher in conventional than in organic vineyards on multiple dates (Figure 10). Similar trends emerged for *Z. rhamni* (vineyard management: F = 6.05; d.f. = 1, 37.5; *p* = 0.018; time: F = 17.00; d.f. = 11, 318; *p* < 0.0001; vineyard management * time: F = 2.56; d.f. = 11, 318; *p* = 0.004; respectively). On some dates, there were more *Z. rhamni* adults in conventional vineyards (Figure 11).

**Figure 12:**
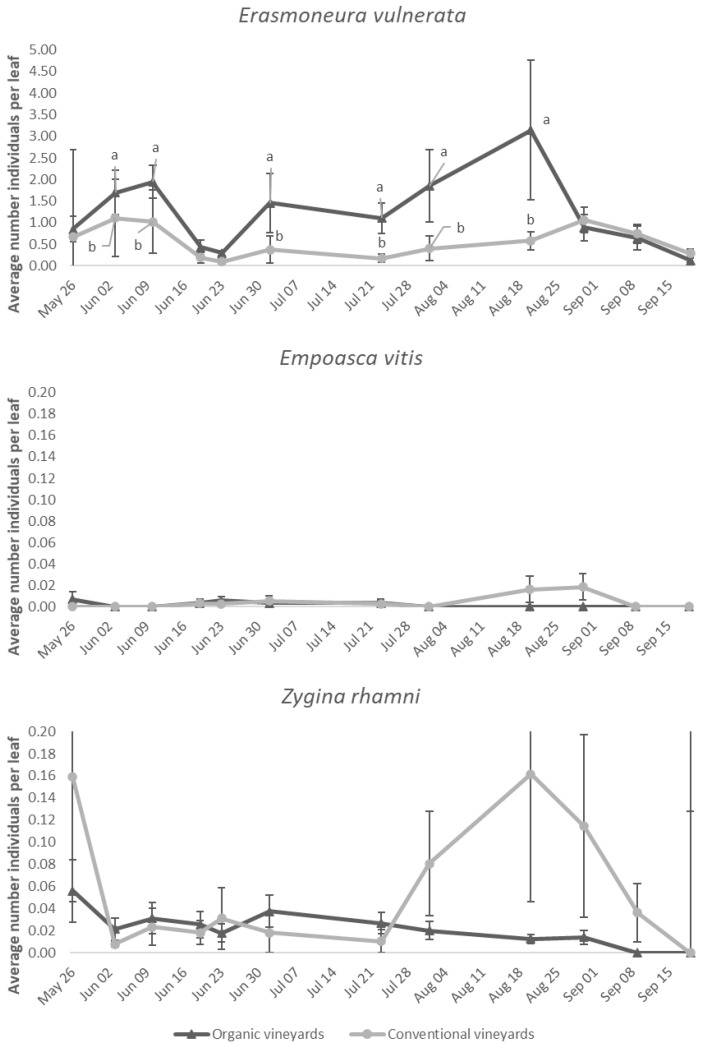
Mean (± std. err.) number of *E. vulnerata*, *E. vitis* and *Z. rhamni* nymphs observed on leaves collected in organic and conventional vineyards. Different letters indicate significant differences at Tukey’s test on least square means (α = 0.05) of the number of nymphs observed on each date.

*Erasmoneura vulnerata* nymph densities were higher in organic vineyards than in conventional vineyards (F = 4.61; d.f. = 1; 18; *p* = 0.046; Figure 12). The effect of time was also significant (F = 3.72; d.f. = 10, 95.1; *p* < 0.001; Figure 11) in contrast to that of the interaction vineyard management * time (F = 1.43; d.f. = 10, 95.1; *p* = 0.18). Vineyard management did not affect *E. vitis* or *Z. rhamni* nymphs (F = 0.66; d.f. = 1, 2.87; *p* = 0.478; F = 1.8; d.f. = 1, 12.7; *p* = 0.203; for *E. vitis* and *Z. rhamni*, respectively, Figure 11). The effect of time and the interaction vineyard management * time were not significant for *E. vitis* (F = 0.98; d.f. = 14, 60.4; *p* = 0.485; F = 1.46; d.f. = 11, 57.7; *p* = 0.171) or *Z. rhamni* (F = 1.78; d.f. = 14, 72.4; *p* = 0.058; F = 1.33; d.f. = 11, 72; *p* = 0.227).

Higher parasitism rates from Mymarids were found in organic vineyards (mean 42%) compared to conventional vineyards (mean 23%) (F = 7.08; d.f. = 1; 19.8; *p* = 0.015). In organic vineyards, the highest parasitism rates were observed in late spring (55.5%) and late summer (54%). The effect of time was significant (F = 2.57; d.f. = 7, 66.1; *p* = 0.021), while the interaction vineyard management * time was not (F = 1.13; d.f. = 7, 66.1; *p* = 0.358). This is likely due to the decline in parasitism rates during mid-summer in both situations.

## 4. Discussion

In their review, Olivier et al. [28] considered *E. vulnerata* to be among the most economically significant leafhoppers occurring in North American vineyards, something that Zimmerman et al. [13], had already reported 16 years ago. In the latter study, which was conducted in Colorado, *E. vulnerata* populations were found to be mixed with that of *Erythroneura ziczac* Walsh, *E. ziczac* being more frequent. Leafhopper (*E. ziczac* in particular) abundance implied the need for insecticide applications to reduce possible damage, likely leading to an effect on population densities. Based on observations made in two subsequent years, authors suggested that, in Colorado, *E. vulnerata* was able to complete two generations per year. To explain why *E. vulnerata* was less abundant than *E. ziczac* in these vineyards, Zimmerman et al. [13] suggested three factors: Colorado’s environmental conditions, the susceptibility of grape cultivars/hybrids, and the behavior of *E. vulnerata* nymphs that colonize upper leaf surfaces which, in turn, leads to increased exposure to natural enemies. Years later, Triapitsyn et al. [29] surveyed the same Colorado sites previously investigated by Zimmerman et al. [13] to shed some light on the communities of egg parasitoids associated with grapevine leafhoppers. Surprisingly, *E. vulnerata* was found to be the dominant species among leafhoppers, while *E. ziczac* was not detected. These contradictory findings suggest that the pest status of *E. vulnerata* in native areas is not defined.

Our results highlight the most recent pest status of *E. vulnerata* in northern Italy. These findings can have serious implications for other Italian regions and European countries (e.g., Switzerland, Slovenia and Serbia) where this pest has been detected more recently. Prior to this study, the damage potential of *E. vulnerata* was a matter of discussion in North America [13,29] and was just hypothesized about on American grapevine cultivars in Europe [17]. Recent reports of *E. vulnerata* outbreaks in commercial *V. vinifera* vineyards in southern Europe suggest a need for investigating factors influencing its potential as a new pest. Previous observations on the pest’s phenology carried out on *Vitis labrusca* in Italy suggested that this species could complete two or three generations per year [17]. The present study strongly supports that *E. vulnerata* completes three generations per year in *V. vinifera* vineyards. In the present study, several *V. vinifera* cultivars (e.g., Cabernet Sauvignon, Merlot, Chardonnay, Pinot gris, Garganega, Trebbiano, Glera) were found to be equally infested, providing no evidence for variable susceptibility among cultivars located on the same farm. Overwintering is a crucial phase for the success of vineyard colonization by *E. vulnerata*. Zimmerman et al. [13] detected overwintering adults near the vineyard margins within the evergreen canopy, inside plant structures, or under organic litter. Initial observations carried out in Northern Italy highlighted the importance of rural buildings or hedgerows near vineyards for overwintering and pest colonization [17]. In the present study, a significant edge effect was found in vineyard colonization by *E. vulnerata* during the spring. In this phase, vineyards were also colonized by the native leafhoppers *E. vitis* and *Z. rhamni*, but their incidence in investigated vineyards was very low. Data suggest the possible role of interspecific competition and the need to investigate this phenomenon in controlled conditions. In a vineyard near those investigated in the present study, high numbers of adult *E. vulnerata* at bud break seriously impacted shoot growth, resulting in the need for control measures.

Among other factors promoting the pest status of *E. vulnerata* in Northern Italy, tolerance to some pesticides has been suggested [19]. In this study, we investigated the effect of vineyard management on the seasonal abundance of *E. vulnerata* and found higher pest population densities in organic vineyards. In Italy and in other European countries, organic vineyards can be managed with copper-based fungicides, sulfur, and a few natural insecticides, mostly pyrethrins. Results strongly suggest that these insecticides are less effective than conventional insecticides in keeping leafhoppers at acceptable density levels. Interestingly, predation and parasitism did not increase significantly in organic vineyards to compensate for the low efficacy of pyrethrins or other natural insecticides. Natural control of *E. vulnerata* and *E. ziczac* by egg parasitoids in Colorado appeared to be negligible (parasitism rate < 2%) [13], while the role of egg parasitoids in newly invaded areas appeared to be promising. The most common egg parasitoid encountered in the present study, *A. parvus*, is frequently associated with *Z. rhamni* in north-eastern Italy [20,30].

Our results have implications for *E. vulnerata* control. Knowledge of the “edge effect” in adult colonization could help growers in early detection of this pest and lead to the adoption of adequate control measures. This is particularly important when high leafhopper numbers infest plants at bud break, imposing a threat on shoot growth. In this regard, based on the information provided in the present study, yellow sticky traps should be placed at the vineyard margins in March. This will provide useful information on adult colonization patterns. Data obtained on natural control by Mymarids suggest properly managing areas surrounding vineyards to preserve alternative hosts that promote Mymarid overwintering [31]. Finally, the limited efficacy of pyrethrins against *E. vulnerata* and their negative side effects on beneficials suggest the need for alternative control measures. Trials have been carried out with kaolin based on the results obtained from other leafhopper species [32]. These trials are showing promising results.

## 5. Conclusions

In conclusion, *E. vulnerata* completes three generations per year in *V. vinifera* vineyards in Northern Italy. Also, there is no apparent difference in susceptibility among the most common cultivars tested in this study. Our study strongly supports an edge effect on *E. vulnerata* vineyard colonization during the spring. This aspect should be considered during the application of modern IPM strategies. Also, future investigations should aim to identify control solutions in organic farms where natural insecticides are not very effective. Moreover, the economic impact of *E. vulnerata* on grapevine yield and quality should be investigated to define economic threshold levels and reduce pesticide use.

## Figures and Tables

**Table 1:** Vineyards considered in studies on the colonization patterns and phenology of *E. vulnerata*. Dots indicate which study was performed in these vineyards.

Year	Vineyard	Management	Locality	Cultivar	Insecticides Used (n° Applicat.)	Colonization Patterns	Phenology
2017	AO	Organic	Alonte	Merlot, Glera,	Pyrethrins (1)	●	●
			45°22′0″ N, 11°25′41″ E	Pinot gris, Incrocio Manzoni 6013			
2017	LO1	Organic	Lonigo	Merlot, Trebbiano T.	Insecticide-free	●	
			45°23′18″ N, 11°23′24″ E				
2017	AC	Conventional	Alonte	Cabernet S., Pinot gris, Glera	Insecticide-free	●	●
			45°21′33.6″ N, 11°26′11.3″ E				
			Lonigo	Garganega,	Insecticide-free	●	
2017	LC	Conventional	45°24′16.2″ N 11°22′32.2″ E	Chardonnay,			
				Merlot			
2018	AO	Organic	Alonte	Merlot, Glera	Insecticide-free	●	
			45°22′38.6″ N 11°24′30.6″ E				
2018	LO2	Organic	Lonigo	Trebbiano T., Cabernet S.	Insecticide-free	●	●
			45°22′41.2″ N 11°24′34.4″ E				
2018	MO	Organic	Monteforte	Garganega	Mineral oil (1)		●
			45°25′5” N, 11°17′4″ E				
2018	LC	Conventional	Lonigo	Garganega	Insecticide-free	●	
2018	MC	Conventional	Monteforte	Trebbiano S.	Insecticide-free	●	
			45°27′16.6″ N, 11°16′59.2″ E				
2019	LO1	Organic	Lonigo	Merlot	Pyrethrins (2)	●	●
2019	MO	Organic	Monteforte	Garganega	Insecticide-free	●	●
2019	AC	Conventional	Alonte	Cabernet S., Glera	Insecticide-free	●	
2019	GC	Conventional	Gambellara	Garganega	Insecticide-free	●	
			45°24′17.9″ N 11°22′31.8″ E				

## References

[1] McAtee W.L. (1920). Key to the nearctic species and varieties of *Erythroneura* (Homoptera; Eupterygidae). Trans. Am. Entomol. Soc..

[2] Johnson D.M. (1935). Leafhoppers of Ohio. Subfamily Typhlocybinae (Homoptera: Cicadellidae). Bull. Ohio Biol. Surv..

[3] Metcalf Z.P. (1968). General Catalogue of the Homoptera. VI. Cicadelloidea. Part 17.

[4] Dmitriev D.A., Dietrich C.H. (2007). Review of the New World *Erythroneurini* (Hemiptera: Cicadellidae: Typhlocybinae). I. Genera *Erythroneura, Erasmoneura, Rossmoneura*, and *Hymetta*. Ill. Nat. Hist. Surv..

[5] Wilson L.T., Barnes M.M., Flaherty D.L., Andris H.L., Leavitt G.M. (1992). Variegated Grape Leafhopper. Grape Pest Management.

[6] Daane K.M., Yokota G.Y., Zheng Y., Hagen K.S. (1996). Inundative release of common green lacewings (Neuroptera: Chrysopidae) to suppress *Erythroneura variabilis* and *E. elegantula*. (Homoptera: Cicadellidae) in vineyards. Environ. Entomol..

[7] Costello M.J. (2008). Regulated deficit irrigation and density of *Erythroneura* spp. (Hemiptera: Cicadellidae) on grape. J. Econ. Entomol..

[8] Dmitriev D.A. 3I Interactive Keys and Taxonomic Databases. Auchenorrhyncha Database Search. http://dmitriev.speciesfile.org.

[9] Robinson W. (1926). The genus *Erythroneura* north of Mexico (Homoptera, Cicadellidae). Sci. Bull. Univ. Kans..

[10] Beamer R.H. (1946). The *Erythroneura* of the vulnerata group (Homoptera—Cicadellidae). J. Kans. Entomol. Soc..

[11] Martinson T.E., Dennehy T.J. (1995). Varietal preferences of *Erythroneura* leafhoppers (Homoptera: Cicadellidae) feeding on grapes in New York. Environ. Entomol..

[12] Paxton D.W., Thorvilson H.G. (1996). Oviposition of three *Erythroneura* species on grape leaves in Western Texas. Southwest. Entomol..

[13] Zimmerman R., Kondratieff B., Nelson E., Sclar C. (1996). The life history of two species of grape leafhoppers on wine grapes in western Colorado. J. Kans. Entomol. Soc..

[14] Duso C., Bressan A., Mazzon L., Girolami V. (2005). First record of the grape leafhopper *Erythroneura vulnerata* Fitch (Hom., Cicadellidae) in Europe. J. Appl. Entomol..

[15] Girolami V., Duso C., Mazzon L., Bressan A. (2006). Nuova cicalina della vite in Italia. Inf. Agric..

[16] Duso C., Moret R., Marchegiani G., Pozzebon A. (2008). Notes on the distribution and the phenology of *Erasmoneura vulnerata* (Fitch) (Homoptera: Cicadellidae) in North-eastern Italy. IOBC/WPRS Bull..

[17] Duso C., Moret R., Manera A., Berto D., Fornasiero D., Marchegiani G., Pozzebon A. (2019). Investigations on the grape leafhopper *Erasmoneura vulnerata* in North-eastern Italy. Insects.

[18] Seljak G. (2011). First record of the Nearctic leafhopper *Erasmoneura vulnerata* (Fitch, 1851) (Hemiptera, Cicadomorpha: Cicadellidae) in Slovenia. Acta Entomol. Slov..

[19] Duso C., Borgo M., Pozzebon A., Mazzon L., Mori N., Pavan F., Fornasiero D., Marchesini E., Martinez-Sañudo I., Zanettin G. (2017). Vite: *Erasmoneura vulnerata*, una minaccia da valutare. Inf. Agric..

[20] Rizzoli A., Battelli R., Conedera M., Jermini M. (2020). First record of *Erasmoneura vulnerata* Fitch, 1851 (Hemiptera, Cicadellidae, Typhlocybinae) in Switzerland. Alp. Entomol..

[21] Šćiban M., Kosovac A. (2020). New records and updates on alien Auchenorrhyncha species in Serbia. Pesticidi i Fitomedicina.

[22] Zanolli P., Pavan F. (2011). Autumnal emergence of *Anagrus* wasps, egg parasitoids of *Empoasca vitis*, from grapevine leaves and their migration towards brambles. Agric. For. Entomol..

[23] Vidano C. (1958). Le cicaline italiane della vite. Hemiptera Typhlocibinae. Boll. Zool. Agr. Bachic..

[24] Patwary M.U., Kenchington E.L., Bird C.J., Zouros E. (1994). The use of random amplified polymorphic DNA markers in genetic studies of the sea scallop *Placopecten magellanicus* (Gmelin, 1791). J. Shellfish Res..

[25] Martinez-Sañudo I., Mazzon L., Simonato M., Avtzis D., Pujade-Villar J., Faccoli M. (2019). Tracking the origin and dispersal of the Asian chestnut gall wasp *Dryocosmus kuriphilus* Yasumatsu (Hymenoptera, Cynipidae) in Europe with molecular markers. Bull. Entomol. Res..

[26] Viggiani G. (2014). On the misidentification of *Anagrus ustulatus* Haliday (Hymenoptera: Mymaridae). Zootaxa.

[27] Mazzon L., Martinez-Sañudo I. (2019). Personal communication.

[28] Olivier C., Vincent C., Saguez J., Galka B., Weintraub P.G., Maixner M. (2012). Leafhoppers and planthoppers: Their bionomics, pathogen transmission and management in vineyards. Arthropod Management in Vineyards.

[29] Triapitsyn S.V., Rugman-Jones P.F., Jeong G., Morse J.G., Stouthamer R. (2010). Morphological and molecular differentiation of the *Anagrus epos* species complex (Hymenoptera: Mymaridae), egg parasitoids of leafhoppers (Hemiptera: Cicadellidae) in North America. Zootaxa.

[30] Zanolli P., Martini M., Mazzon L., Pavan F. (2016). Morphological and Molecular identification of *Anagrus ‘atomus’* Group (Hymenoptera: Mymaridae) individuals from different geographic areas and plant hosts in Europe. J. Insect Sci..

[31] Ponti L., Ricci C., Veronesi F., Torricelli R. (2005). Natural hedges as an element of functional biodiversity in agroecosystems: The case of a Central Italy vineyard. Bull. Insectol..

[32] Tacoli F., Pavan F., Cargnus E., Tilatti E., Pozzebon A., Zandigiacomo P. (2017). Efficacy and mode of action of kaolin in the control of *Empoasca vitis* and *Zygina rhamni* (Hemiptera: Cicadellidae) in vineyards. J. Econ. Entomol..

