# Correction: Duso C.; et al. Colonization Patterns, Phenology and Seasonal Abundance of the Nearctic Leafhopper *Erasmoneura vulnerata* (Fitch), a New Pest in European Vineyards. *Insects* 2020, *11*, 731

**DOI:** 10.3390/insects12010059

**Published:** 2021-01-12

**Authors:** Carlo Duso, Giulia Zanettin, Pamela Gherardo, Giulia Pasqualotto, Damiano Raniero, Filippo Rossetto, Paola Tirello, Alberto Pozzebon

**Affiliations:** Department of Agronomy, Food, Natural Resources, Animals and Environment, University of Padova, Viale dell’Università 16, Agripolis, 35020 Legnaro, Padova, Italy; giuliazanettin22@gmail.com (G.Z.); pamela.gherardo@studenti.unipd.it (P.G.); giulia.pasqualotto.2@studenti.unipd.it (G.P.); damiano.raniero@studenti.unipd.it (D.R.); filippo.rossetto.1@studenti.unipd.it (F.R.); paola.tirello@unipd.it (P.T.); alberto.pozzebon@unipd.it (A.P.)

It has recently come to our attention that there were some mistakes in legends and figures reported in our study [1]. In Figure 5, Figure 6, Figure 7, Figure 8, Figure 9 and Figure 10, “Istar” was reported instead of “Instar”. Moreover, the legend of Figure 9 was corrected as follows: “The phenology of *E. vulnerata* in LO1 vineyard in the growing season of 2019. Arrows indicate pyrethrins treatments.” and the legend of Figure 10 was corrected as follows: “The phenology of *E. vulnerata* in MO vineyard in the growing season of 2019”. The corrected figures and legends are reported below. 

In the text, on page 10, Section 3.2.3, “2019”, Figure 9 and Figure 10 should be cited instead of Figure 8 and Figure 9.

The authors apologize for any inconvenience caused and state that the scientific conclusions are unaffected. The original article has been updated.

## Figures and Tables

**Figure 5:**
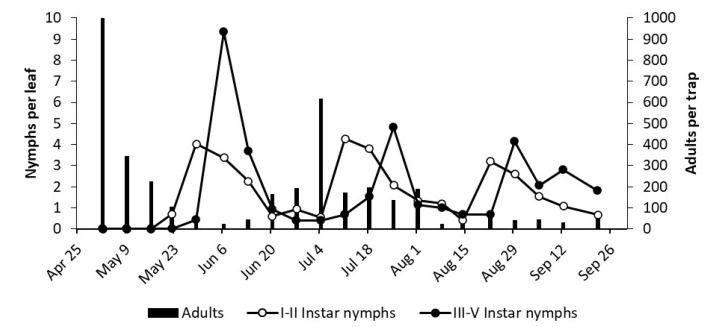
The phenology of *E. vulnerata* in AC vineyard in the growing season of 2017.

**Figure 6:**
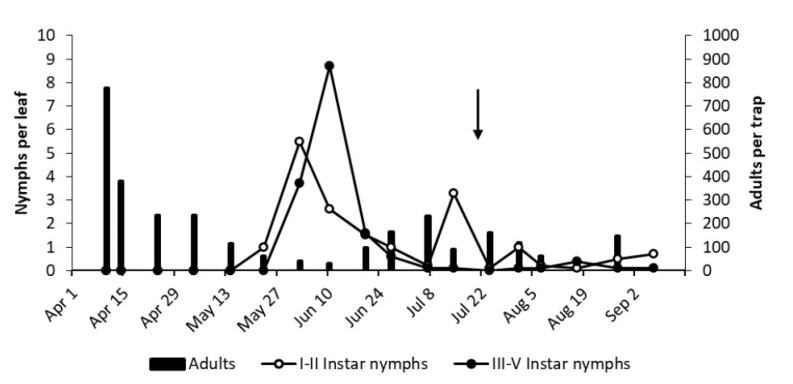
The phenology of *E. vulnerata* in AO vineyard in the growing season of 2017. Arrow indicates pyrethrins treatment.

**Figure 7:**
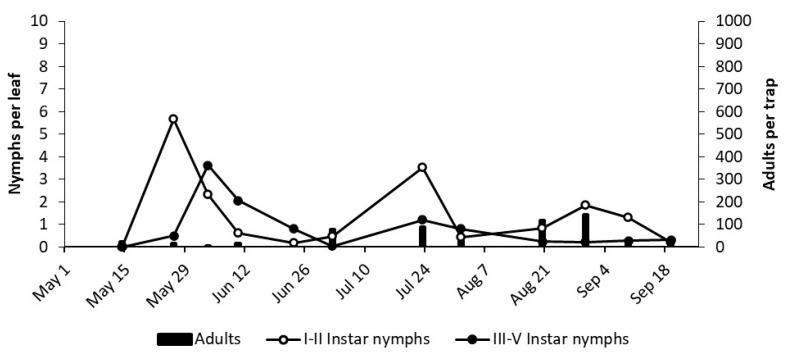
The phenology of *E. vulnerata* in MO vineyard in the growing season of 2018.

**Figure 8:**
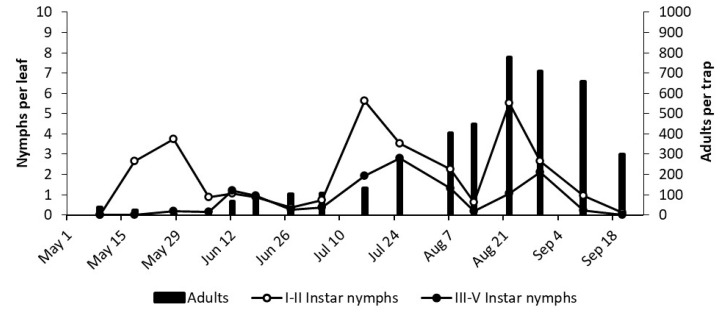
The phenology of *E. vulnerata* in LO2 vineyard in the growing season of 2018.

**Figure 9:**
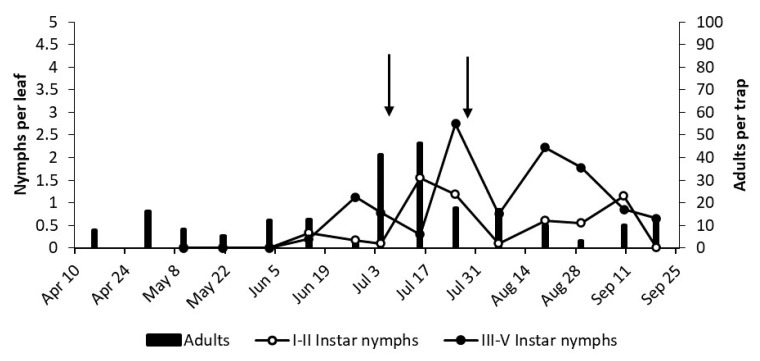
The phenology of *E. vulnerata* in LO1 vineyard in the growing season of 2019. Arrows indicate pyrethrins treatments.

**Figure 10:**
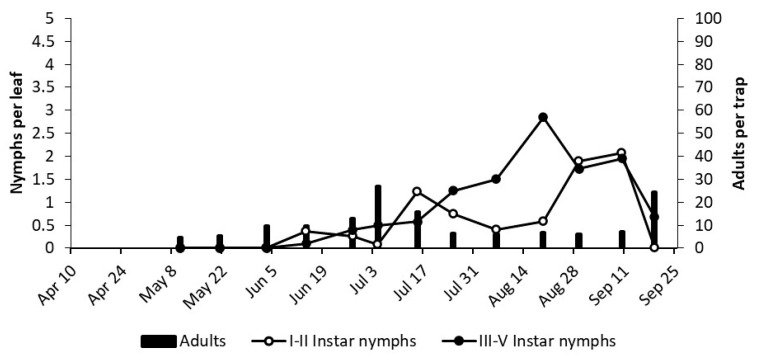
The phenology of *E. vulnerata* in MO vineyard in the growing season of 2019.
